# *catena*-Poly[copper(II)-bis­(μ-3-cyano-2-hydroxy­propionato)-κ^3^ *N*:*O* ^1^,*O* ^2^;κ^3^ *O* ^1^,*O* ^2^:*N*-copper(II)]

**DOI:** 10.1107/S1600536810007129

**Published:** 2010-03-03

**Authors:** Ji-Dong Wang, Shu-Min Han

**Affiliations:** a College of Environmental and Chemical Engineering, Yanshan University, Qinhuangdao 066004, People’s Republic of China; b College of Information Technology and Engineering, Yanshan University, Qinhuangdao 066004, People’s Republic of China; c State Key Laboratory of Metastable Materials Science and Technology, Yanshan University, Qinhuangdao 066004, People’s Republic of China

## Abstract

The title compound, [Cu(C_4_H_4_NO_3_)_2_]_*n*_, exhibits a double-chain structure extending along [100]. The Cu^II^ atom, lying on an inversion center, is coordinated by two cyano N atoms from two 3-cyano-2-hydroxy­propionate ligands and two hydr­oxy O atoms and two carboxyl­ate O atom from two other two ligands in a distorted octa­hedral geometry. Inter­molecular C—H⋯O and O—H⋯O hydrogen bonds connect the chains into a three-dimensional structure.

## Related literature

For the synthesis and studies of β-hydroxy­nitriles, see: Conti *et al.* (2003 ▶); Seo *et al.* (1994 ▶). For related structures, see: Klein *et al.* (1982 ▶); Wang *et al.* (2009 ▶).
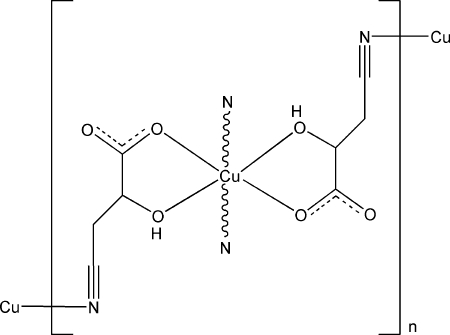


## Experimental

### 

#### Crystal data

[Cu(C_4_H_4_NO_3_)_2_]
*M* *_r_* = 291.71
Monoclinic, 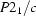

*a* = 6.3704 (7) Å
*b* = 8.4382 (10) Å
*c* = 10.0412 (12) Å
β = 104.492 (2)°
*V* = 522.59 (11) Å^3^
*Z* = 2
Mo *K*α radiation
μ = 2.11 mm^−1^
*T* = 293 K
0.28 × 0.19 × 0.12 mm

#### Data collection

Bruker SMART APEX CCD diffractometer
Absorption correction: multi-scan (*SADABS*; Sheldrick, 1996 ▶) *T*_min_ = 0.624, *T*_max_ = 0.776
2803 measured reflections
1031 independent reflections
973 reflections with *I* > 2σ(*I*)
*R*_int_ = 0.017

#### Refinement

*R*[*F* ^2^ > 2σ(*F* ^2^)] = 0.023
*wR*(*F* ^2^) = 0.064
*S* = 1.11
1031 reflections
83 parameters
1 restraint
H atoms treated by a mixture of independent and constrained refinement
Δρ_max_ = 0.32 e Å^−3^
Δρ_min_ = −0.27 e Å^−3^

### 

Data collection: *SMART* (Bruker, 2007 ▶); cell refinement: *SAINT* (Bruker, 2007 ▶); data reduction: *SAINT*; program(s) used to solve structure: *SHELXS97* (Sheldrick, 2008 ▶); program(s) used to refine structure: *SHELXL97* (Sheldrick, 2008 ▶); molecular graphics: *SHELXTL* (Sheldrick, 2008 ▶) and *Mercury* (Macrae *et al.*, 2006 ▶); software used to prepare material for publication: *SHELXTL*.

## Supplementary Material

Crystal structure: contains datablocks global, I. DOI: 10.1107/S1600536810007129/hy2278sup1.cif

Structure factors: contains datablocks I. DOI: 10.1107/S1600536810007129/hy2278Isup2.hkl

Additional supplementary materials:  crystallographic information; 3D view; checkCIF report

## Figures and Tables

**Table 1:** Selected bond lengths (Å)

Cu1—O2	1.9159 (12)
Cu1—O1	1.9579 (11)
Cu1—N1^i^	2.545 (2)

Symmetry code: (i) 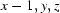.

**Table 2:** Hydrogen-bond geometry (Å, °)

*D*—H⋯*A*	*D*—H	H⋯*A*	*D*⋯*A*	*D*—H⋯*A*
C1—H1⋯O2^ii^	0.98	2.54	3.240 (2)	128
O1—H1*W*⋯O3^ii^	0.83 (2)	1.75 (2)	2.560 (2)	165 (4)

Symmetry code: (ii) 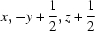.

**Fig. 1.**
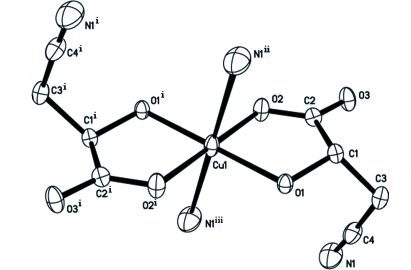
The structure of the title compound. Displacement ellipsoids are drawn at the 30% probability level. H atoms have been omitted for clarity. [Symmetry codes: (i) 1-x, -y, -z; (ii) x-1, y, z; (iii) 2-x, -y, -z.]

**Fig. 2.**
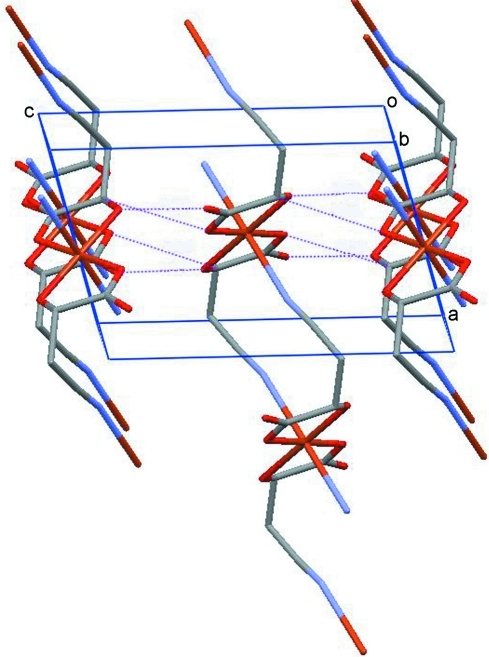
The one-dimensional double-chain in the title compound. Dashed lines denote hydrogen bonds.

## supplementary crystallographic information

### Comment

β-Hydroxynitriles are potentially important intermediates in the synthesis of complex organic compounds (Seo *et al.*, 1994). The study of coordination polymers with β-hydroxynitrile is rarely reported according to Cambridge Structural Database. Herein we report the structure of the title compound.

In the title compound, the Cu^II^ atom, lying on an inversion center, is six-coordinated in a distorted octahedral geometry defined by two carboxylate O atoms and two hydroxy O atoms in the equatorial plane and two N atoms from the cyano groups in the axial positions (Table 1 and Fig. 1). Weak coordination between the Cu^II^ atom and the N atoms is indicated by a Cu—N distance of 2.545 (2) Å, due to Jahn-Teller effects. The bond lengths and angles are in normal ranges (Klein *et al.*, 1982; Wang *et al.*, 2009). Adjacent Cu^II^ centers are bridged by two ligands, forming a double-chain structure, which is further extended by intermolecular C—H···O and O—H···O hydrogen bonds (Table 2) into a three-dimentional supramolecular structure.

### Experimental

2-Isoxazoline-3,5-dicarboxylic acid was synthesized according to the previously reported procedure (Conti *et al.*, 2003). 3-Cyano-2-hydroxypropionic acid was obtained from 2-isoxazoline-3,5-dicarboxylic acid by selective cleavage of the N—O bond and decarboxylation under basic condition (Seo *et al.*, 1994). A solution of Cu(NO_3_)_2_.3H_2_O (0.048 g, 0.2 mmol) in H_2_O (4 ml) was added to a solution of 3-cyano-2-hydroxypropionic acid (0.046 g, 0.4 mmol) in H_2_O (8 ml), then aqueous triethylamine (0.07 ml) was added dropwise to the above solution accompanied with stirring. The mixture was flitered and placed at room temperature. Blue block crystals of the title compound were obtained in three days (yield 0.046 g, 78% based on Cu).

### Refinement

C-bound H atoms were positioned geometrically and refined as riding atoms, with C—H = 0.98 (CH) and 0.97 (CH_2_) Å and with *U*_iso_(H) = 1.2*U*_eq_(C). The hydroxy H atom was found in a difference Fourier map and refined isotropically.

### Crystal data

**Table:**

[Cu(C_4_H_4_NO_3_)_2_]	*F*(000) = 294
*M**_r_* = 291.71	*D*_x_ = 1.854 Mg m^−^^3^
Monoclinic, *P*2_1_/*c*	Mo *K*α radiation, λ = 0.71073 Å
Hall symbol: -P 2ybc	Cell parameters from 2150 reflections
*a* = 6.3704 (7) Å	θ = 3.2–26.0°
*b* = 8.4382 (10) Å	µ = 2.11 mm^−^^1^
*c* = 10.0412 (12) Å	*T* = 293 K
β = 104.492 (2)°	Block, blue
*V* = 522.59 (11) Å^3^	0.28 × 0.19 × 0.12 mm
*Z* = 2	

### Data collection

**Table:**

Bruker SMART APEX CCD diffractometer	1031 independent reflections
Radiation source: sealed tube	973 reflections with *I* > 2σ(*I*)
graphite	*R*_int_ = 0.017
φ and ω scans	θ_max_ = 26.0°, θ_min_ = 3.2°
Absorption correction: multi-scan (*SADABS*; Sheldrick, 1996)	*h* = −5→7
*T*_min_ = 0.624, *T*_max_ = 0.776	*k* = −9→10
2803 measured reflections	*l* = −12→12

### Refinement

**Table:**

Refinement on *F*^2^	Primary atom site location: structure-invariant direct methods
Least-squares matrix: full	Secondary atom site location: difference Fourier map
*R*[*F*^2^ > 2σ(*F*^2^)] = 0.023	Hydrogen site location: inferred from neighbouring sites
*wR*(*F*^2^) = 0.064	H atoms treated by a mixture of independent and constrained refinement
*S* = 1.11	*w* = 1/[σ^2^(*F*_o_^2^) + (0.0477*P*)^2^ + 0.2506*P*] where *P* = (*F*_o_^2^ + 2*F*_c_^2^)/3
1031 reflections	(Δ/σ)_max_ < 0.001
83 parameters	Δρ_max_ = 0.32 e Å^−^^3^
1 restraint	Δρ_min_ = −0.27 e Å^−^^3^

### Fractional atomic coordinates and isotropic or equivalent isotropic displacement parameters (Å^2^)

**Table:**

	*x*	*y*	*z*	*U*_iso_*/*U*_eq_	
Cu1	0.5000	0.0000	0.0000	0.03130 (14)	
N1	1.1980 (3)	0.1558 (3)	0.0646 (2)	0.0635 (6)	
O1	0.69000 (19)	0.11889 (14)	0.15024 (11)	0.0264 (3)	
H1W	0.671 (4)	0.117 (3)	0.2292 (14)	0.057 (7)*	
O2	0.5444 (2)	0.17730 (15)	−0.10898 (11)	0.0364 (3)	
O3	0.6908 (2)	0.41604 (15)	−0.09635 (12)	0.0384 (3)	
C1	0.7140 (3)	0.28079 (19)	0.11473 (16)	0.0256 (3)	
H1	0.6192	0.3473	0.1545	0.031*	
C2	0.6441 (3)	0.2934 (2)	−0.04252 (16)	0.0274 (4)	
C3	0.9491 (3)	0.3336 (2)	0.17056 (18)	0.0345 (4)	
H3A	0.9641	0.4433	0.1454	0.041*	
H3B	0.9880	0.3266	0.2701	0.041*	
C4	1.0950 (3)	0.2340 (3)	0.1150 (2)	0.0419 (5)	

### Atomic displacement parameters (Å^2^)

**Table:**

	*U*^11^	*U*^22^	*U*^33^	*U*^12^	*U*^13^	*U*^23^
Cu1	0.0435 (2)	0.0282 (2)	0.02069 (19)	−0.01409 (12)	0.00516 (14)	0.00079 (10)
N1	0.0422 (10)	0.0728 (14)	0.0791 (14)	−0.0038 (10)	0.0222 (10)	−0.0190 (12)
O1	0.0346 (6)	0.0251 (6)	0.0203 (5)	−0.0059 (5)	0.0080 (5)	−0.0001 (4)
O2	0.0537 (8)	0.0323 (7)	0.0217 (6)	−0.0152 (6)	0.0065 (5)	0.0008 (5)
O3	0.0626 (9)	0.0272 (7)	0.0284 (6)	−0.0107 (6)	0.0170 (6)	0.0016 (5)
C1	0.0333 (9)	0.0228 (8)	0.0232 (7)	−0.0030 (6)	0.0116 (6)	−0.0017 (6)
C2	0.0327 (8)	0.0276 (8)	0.0244 (8)	−0.0008 (7)	0.0120 (7)	0.0001 (6)
C3	0.0385 (10)	0.0351 (9)	0.0306 (8)	−0.0119 (8)	0.0099 (7)	−0.0069 (7)
C4	0.0307 (9)	0.0476 (11)	0.0465 (11)	−0.0109 (9)	0.0081 (8)	−0.0063 (9)

### Geometric parameters (Å, °)

**Table:**

Cu1—O2	1.9159 (12)	O3—C2	1.238 (2)
Cu1—O1	1.9579 (11)	C1—C3	1.528 (2)
Cu1—N1^i^	2.545 (2)	C1—C2	1.533 (2)
N1—C4	1.135 (3)	C1—H1	0.9800
O1—C1	1.4298 (19)	C3—C4	1.464 (3)
O1—H1W	0.832 (10)	C3—H3A	0.9700
O2—C2	1.264 (2)	C3—H3B	0.9700
			
O2^ii^—Cu1—O2	180.0	C3—C1—C2	111.28 (14)
O2^ii^—Cu1—O1	96.39 (5)	O1—C1—H1	109.3
O2—Cu1—O1	83.62 (5)	C3—C1—H1	109.3
O2—Cu1—O1^ii^	96.38 (5)	C2—C1—H1	109.3
O1—Cu1—O1^ii^	179.999 (1)	O3—C2—O2	124.17 (15)
O1—Cu1—N1^i^	84.25 (6)	O3—C2—C1	117.92 (14)
O2—Cu1—N1^i^	88.35 (6)	O2—C2—C1	117.90 (14)
O1^ii^—Cu1—N1^i^	95.74 (6)	C4—C3—C1	110.51 (15)
O2^ii^—Cu1—N1^i^	91.65 (6)	C4—C3—H3A	109.5
C1—O1—Cu1	112.43 (9)	C1—C3—H3A	109.5
C1—O1—H1W	108.0 (19)	C4—C3—H3B	109.5
Cu1—O1—H1W	120.9 (19)	C1—C3—H3B	109.5
C2—O2—Cu1	115.49 (10)	H3A—C3—H3B	108.1
O1—C1—C3	110.09 (14)	N1—C4—C3	175.7 (2)
O1—C1—C2	107.56 (12)		

Symmetry codes: (i) *x*−1, *y*, *z*; (ii) −*x*+1, −*y*, −*z*.

### Hydrogen-bond geometry (Å, °)

**Table:**

*D*—H···*A*	*D*—H	H···*A*	*D*···*A*	*D*—H···*A*
C1—H1···O2^iii^	0.98	2.54	3.240 (2)	128
O1—H1W···O3^iii^	0.83 (2)	1.75 (2)	2.560 (2)	165 (4)

Symmetry codes: (iii) *x*, −*y*+1/2, *z*+1/2.
